# Mussel-Inspired Surface Modification of α-Zirconium Phosphate Nanosheets for Anchoring Efficient and Reusable Ultrasmall Au Nanocatalysts

**DOI:** 10.3390/nano12193339

**Published:** 2022-09-25

**Authors:** Limiao Lin, Yi Wen, Lixi Li, Ying Tan, Peng Yang, Yaoheng Liang, Yisheng Xu, Huawen Hu, Yonghang Xu

**Affiliations:** 1 School of Environmental and Chemical Engineering, Foshan University, Foshan 528000, China; 2 School of Materials Science and Hydrogen Energy, Foshan University, Foshan 528000, China

**Keywords:** ternary composite catalyst, powerful functionalities, metal nanocatalysts, 2D structure, catalytic efficiency, reusability

## Abstract

The shortage of powerful functionalities on scalable α-zirconium phosphate (ZrP) materials blocks the facile preparation of highly dispersed and immobilized metal nanocatalysts. We herein present a mild and facile mussel-inspired strategy based on polydopamine (PDA) for the surface modification of ZrP, and hence, the generation of powerful functionalities at a high density for the straightforward reduction of chloroauric acid to Au nanoparticles (AuNPs) and the immobilization of AuNPs. The resulting ternary ZrP@PDA/Au exhibited ultra-small AuNPs with a particle size of around 6.5 nm, as estimated based on TEM images. Consequently, the ZrP@PDA/Au catalyst showed significant activity in the catalytic conversion of 4-nitrophenol (4NP) to 4-aminophenol (4AP), a critical transformation reaction in turning the hazard into valuable intermediates for drug synthesis. The PDA was demonstrated to play a critical role in the fabrication of the highly efficient ZrP@PDA/Au catalyst, far outperforming the ZrP/Au counterpart. The turnover frequency (TOF) achieved by the ZrP@PDA/Au reached as high as 38.10 min^−1^, much higher than some reported noble metal-based catalysts. In addition, the ZrP@PDA/Au showed high stability and reusability, of which the catalytic efficiency was not significantly degraded after prolonged storage in solution.

## 1. Introduction

Environmental pollution and energy shortage issues call for new functional materials to remediate the environment and avoid energy depletion [1,2,3,4]. Researchers have explored feasible technologies based on functional materials, such as adsorbents [5], electrocatalysts [6], photocatalysts [7], flocculants [8], Fenton reagents [9], and even microbes [10], to address environmental pollution issues. Most functional material-based methods are applied to remediate the environment by enrichment, flocculation, oxidation, and biodegradation. The adsorption, coagulation, and flocculation treatments still leave behind pollutants, and post-treatments are thus required with additional cost and high possibilities of secondary pollution [11,12]. On the other hand, oxidation and biodegradation may radically degrade pollutants [13,14,15,16,17]. Nevertheless, some organic pollutants can be reused as a valuable resource through conversion to beneficial organics for useful applications rather than complete degradation. For example, 4-nitrophenol (4NP) has captured significant attention due to its carcinogenic, mutagenic, and recalcitrant nature; nevertheless, the catalytic reduction of 4NP can help obtain a beneficial reaction product, that is, 4-aminophenol (4AP), a critical immediate for many practical drug production [18].

To realize the conversion of 4NP to 4AP, researchers have explored various metal-based [19,20] and metal-free [21,22] catalysts. While the activities of metal-free catalysts are still far from satisfactory for practical applications [23], metal-based catalysts (especially noble metal-based ones) may be more promising in practical implementation due to their high-density active sites [24]. However, the high cost and scarcity of noble metals require a rational design strategy to minimize the utilization of the noble metals. Finding a low-cost support material is one of the most promising ways to minimize noble metal usage. 

Thus, a wide range of materials has been explored as noble metal supports, such as SiO_2_ [25], graphene [26], carbon nanotubes [27], Al_2_O_3_ [28], Fe_3_O_4_ [29], polymers [30], and a combination thereof [31]. However, most of these support materials lack sufficient binding sites for noble metals, thus causing the resulting noble metal particles to bear a large size, which is not desirable for obtaining highly efficient metal-based catalysts. This is due to the low content of exposed active sites on the surface of oversized metal particles while accommodating large quantities of inaccessible metal atoms within the bulk. 

To reduce the particle size, one should consider modifying the surface of the support materials to provide abundant binding sites for noble nanoparticles. On the other hand, some carbon-based materials (such as graphene and CNTs) have exhibited a significant ability to carry noble metal species due to their additional functions (e.g., electron-transferring ability); their incorporation can facilitate the redox reactions between the reducing agent and 4NP. However, the high-cost issues of graphene and CNTs are still not resolved, indicating that lower-cost materials should be explored as alternatives. 

Similar to graphene, α-zirconium phosphate (ZrP) is also within the family of two-dimensional (2D) materials. ZrP is easier to prepare with a lower cost and higher biocompatibility compared to the expensive graphene and derivatives with potential toxicity to living organisms [32]. Consequently, ZrP has also been intensively investigated as a support material of active components due to their unique two-dimensional (2D) structure, surface properties, and application potentials in the fabrication of photocatalysts [33], electrocatalysts [34], catalysts [18], and other functional materials [35], as well as in the immobilization of antibacterial agents [36]. Nevertheless, since ZrP alone is insufficient to be a good support material for advanced catalyst fabrication due to the lack of powerful functional groups capable of realizing chemical transformations, it is thus promising to modify the ZrP surface to incorporate additional functionalities and abundant complexation sites. Mussel-inspired chemical routes have been widely explored to modify support material surfaces and fabricate a vast range of metal-based catalytic materials for various critical transformation reactions. Among other mild reducing agents (e.g., biomass extracts, vitamins, and amino acids), mussel-inspired polydopamine is capable of the efficient conversion of noble metal-based acids and salts into metal nanoparticles since the noble metal ions are prone to be reduced without the need for strong and toxic reducing agents. 

In these contexts, we present the first report regarding the mussel-inspired modification of ZrP nanosheets before the straightforward deposition of noble metal nanoparticles. The mussel-inspired modification endowed the ZrP surface to be uniformly coated with a polydopamine (PDA) layer and rendered the subsequent facile decoration of noble metal nanoparticles (in this case, Au, as a typical noble metal) without the need for additional reducing agents. The catechol and amine groups were reported to be capable of converting Au metal ions into zero-valent Au nanoparticles (AuNPs). The critical role of PDA in the fabrication of the highly efficient and reusable Au-based composite catalyst was investigated. The structure of the typical ternary ZrP@PDA/Au and its catalytic performance in converting 4NP to 4AP were clarified. The present work paves the way for the production of low-cost functional support for efficiently dispersing and immobilizing ultra-small noble metal nanoparticles for promoting the utilization efficiency of noble metals. 

## 2. Experiment

### 2.1. Materials

Phosphoric acid (85%), HAuCl_4_ (30 w% in water), and ZrOCl_2_·8H_2_O were obtained from Sigma-Aldrich (Sigma-Aldrich, Shanghai, China). Tris(hydroxymethyl) aminomethane (Tris), dopamine hydrochloride, KOH, NBu_4_OH (TBAH), 4-nitrophenol (4NP), and sodium borohydride (NaBH_4_) were purchased from Aladdin (Aladdin, Shanghai, China). 

### 2.2. Characterizations

X-ray photoelectron spectroscopy (XPS) was operated on a Thermo Fisher Scientific (Waltham, MA, USA) K-Alpha^+^ spectrometer. UV-Vis absorption spectra were detected with a Shimadzu spectrophotometer (UV-1800, Shimadzu, Kyoto, Japan). Fourier transform infrared (FTIR) spectra were measured using a Shimadzu IRAffinity-1S spectroscope (Shimadzu, Kyoto, Japan) in the KBr testing mode. X-ray diffraction (XRD) patterns ranging from 10° to 80° were collected with a Bruker powder diffractometer (Bruker, Karlsruhe, Germany). High-resolution transmission electron microscopy (HRTEM) was observed using an FEI Talos F200s microscope (FEI, Hillsboro, OR, USA) with a Super-X EDS detector. Scanning electron microscopy (SEM) images were captured using an FEI Quanta 400 microscope (FEI, Hillsboro, OR, USA). An inductively coupled plasma optical emission spectrometer (ICP-OES), Agilent 720ES (Agilent, Palo Alto, CA, USA), was employed to determine the accurate gold content in the catalytic dispersion.

### 2.3. Preparation of ZrP@PDA/Au Nanocatalyst

The layered ZrP microcrystals were synthesized using ZrOCl_2_·8H_2_O and H_3_PO_4_ (6.0 M) according to the literature [37]. The layered ZrP was exfoliated into unilaminar nanosheets by TBAH in water to obtain a transparent solution of ZrP nanosheets (0.1 wt.%) [18,38]. The ZrP@PDA/Au nanocatalyst was fabricated according to our previous work [18,39]. First, a mixture of Tris (0.05 M, 2.5 mL), dopamine (0.2 wt.%, 1 mL), and a dispersion of ZrP nanosheets (2.0 mL) was stirred overnight at room temperature in the open air to obtain the black ZrP@PDA. Second, a diluted HAuCl_4_ (11.77 mM) was prepared and then treated with a KOH solution (0.335 mol/L) to a given pH value. Third, the ZrP@PDA (3.0 mL) and a diluted HAuCl_4_ solution (2.0 mL) with the given pH value were mixed and stirred for 6 h, producing a black ZrP@PDA/Au nanocatalyst dispersion. For comparison, the ZrP/Au and PDA/Au composites were fabricated via the above procedures for ZrP@PDA/Au preparation except without using dopamine and ZrP nanosheets, respectively.

### 2.4. Catalytic Reduction of 4-Nitrophenol

The ZrP@PDA/Au catalyst was employed to convert 4NP to 4AP at room temperature. The above black ZrP@PDA/Au dispersion (0.2 mL) was diluted to 50 mL in a volumetric flask to obtain another colorless and transparent catalytic dispersion for the reduction reaction. The accurate Au content of this diluted catalytic dispersion was determined to be 1.33 μg/mL by ICP-OES. Typically, 4NP (3 mL, 0.24 mM), NaBH_4_ (3 mL, 76 mM), and a catalyst dispersion (400 μL) were mixed, and an aliquot of the mixed solution was rapidly introduced into a cuvette. The reaction was monitored in a real-time manner by UV-Vis spectroscopy with an interval of 1 min.

The reusability experiments were performed based on our previous report [18], and five cycles of repeated usage were considered. The concentrations of the reactants and catalyst in the 40 mL reaction solution were identical to the above typical procedures. UV-Vis absorption spectroscopy was employed to probe the conversion of 4NP to 4AP in each cycle. 

## 3. Results and Discussion 

**Figure 1:**
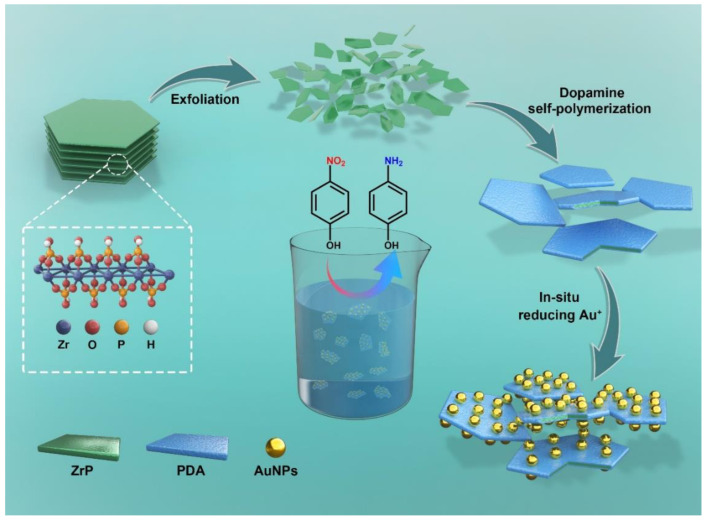
Schematic illustration of the preparation of the ZrP@PDA/AuNP catalyst for the conversion of 4NP to 4AP based on a mussel-inspired strategy.

The monolayer ZrP nanosheets were first prepared via facile hydrothermal reactions and surfactant-assisted water phase exfoliation. The prepared ZrP nanosheets were subsequently adopted as a support material for efficient catalyst synthesis. Before being used as a support, the ZrP was modified by mussel-inspired chemistry based on PDA. The decoration of AuNPs onto the PDA-modified ZrP sheets proceeded with a facile solution reaction using chloroauric acid as an AuNP precursor. The resulting ZrP@PDA/AuNP sample was then applied to the critical chemical transformation reaction (i.e., conversion of 4NP to 4AP). The main procedures adopted in this study are presented in Figure 1. 

**Figure 2:**
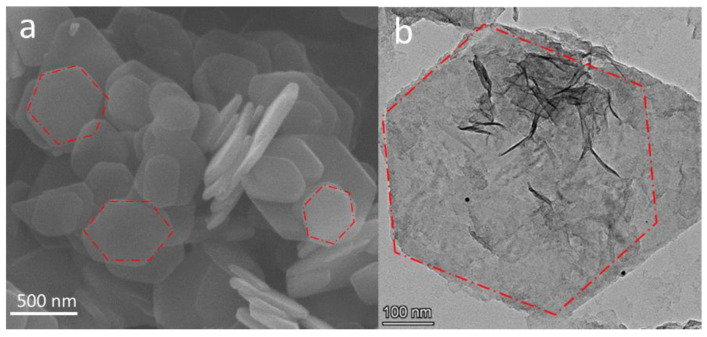
(**a**) SEM image of crystalline ZrP powder, and (**b**) TEM image showing the hexagonal ZrP nanosheets of ZrP@PDA.

**Figure 3:**
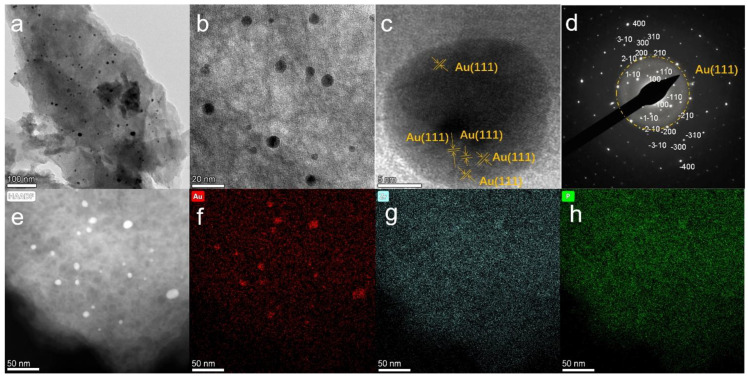
(**a**–**c**) HRTEM images, (**d**) TEM diffraction patterns, (**e**) HAADF image, and the corresponding EDS maps for (**f**) Au, (**g**) Zr, and (**h**) P elements of the prepared ZrP@PDA/Au.

The morphologies of the prepared ZrP and its PDA-modified counterpart are shown in Figure 2a and Figure 2b, respectively. The ZrP exhibited a well-resolved hexagonal sheet structure. The modification of the ZrP by mussel-inspired chemistry based on PDA did not alter the morphology of the ZrP nanosheets, revealing that the PDA homogenously encapsulated the ZrP surface with a nanoscale thickness, which was also reported for PDA coated on graphene materials [19]. Such a nano-thin PDA layer can provide powerful functionalities (such as catechol and amine groups) for the straightforward reduction of chloroauric acid into AuNPs and, meanwhile, the immobilization of AuNPs. The uniform decoration of AuNPs on the ZrP@PDA surface is well demonstrated in Figure 3 and resulted from homogenous binding sites endowed by a nano-thin PDA layer uniformly coated on the ZrP surface. The particle size of the AuNPs on the ZrP@PDA surface can be estimated to be approximately 6.5 nm based on the statistics of the particles displayed in Figure 3a,b. Such an ultra-small AuNP formed is evidence of the numerous anchoring sites provided by PDA. This result also reveals that the PDA is a fascinating material for the modification of ZrP and the straightforward conversion of Au ions into AuNPs. The HRTEM image of an AuNP shown in Figure 3c presents the crystalline fringes of the Au phase, and the adjacent lattice fringe distance was measured to be 0.24 nm, which can be indexed to the Au(111) facets. The SAED pattern of ZrP@PDA/Au shows the single crystal of the ZrP substrate and polycrystalline AuNPs, as evidenced by the patterned bright spots and dispersed rings corresponding to crystalline ZrP and AuNPs, respectively. The SAED index confirms that the ZrP crystal growth proceeded in the (100) direction (Figure 3d) [40]. This finding might be attributed to the preferred growth of the ZrP crystals due to the selective adsorption of the chloride ions on the (001) crystal planes with zirconium atoms exposed, forming zirconium-Cl complexes. The homogeneous elemental dispersion of Au, Zr, and P within the ZrP/PDA/Au matrix can be proven by the HAADF image and the corresponding EDS mapping images shown in Figure 3e–h.

**Figure 4:**
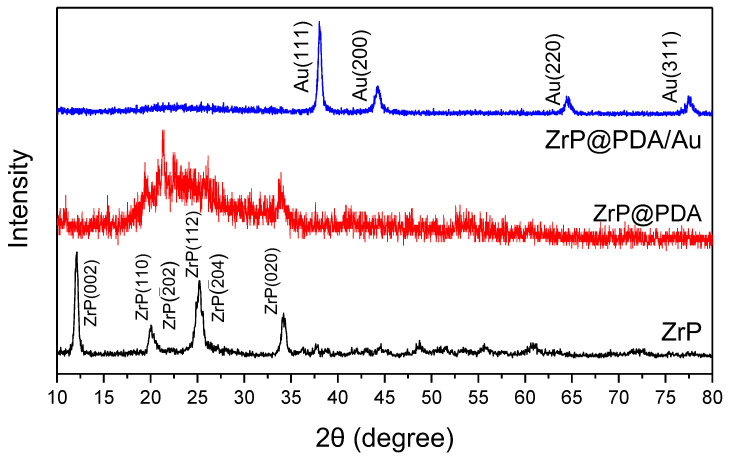
XRD patterns of the ZrP, ZrP@PDA, and ZrP@PDA/Au samples.

XRD patterns of the typical ZrP, ZrP@PDA, and ZrP@PDA/Au samples are presented in Figure 4. The high crystallinity of ZrP can be verified by the sharp diffraction planes (002), (110), (2(-)02), (112), (2(-)04), and (020) [40]. After the PDA modification of ZrP, the XRD pattern underwent a noticeable change, with the sharp peaks depressed due to the amorphous PDA polymer incorporation. The bump centered at approximately 22° corresponds to the PDA composition. Thus, the XRD patterns can sufficiently support the formation of a PDA coating on the ZrP surface. The further decoration of AuNPs on the ZrP@PDA can be evidenced by the appearance of characteristic diffraction bands related to the face-centered cubic (fcc) Au (corresponding to the (111), (200), (220), and (311) crystallographic planes), thus proving the successful conversion of Au ions into AuNPs. The (111) plane diffraction peak was adopted to calculate the crystallite size of the AuNPs based on the Scherrer equation, which was around 17 nm, larger than that estimated from the TEM image, where the AuNPs were highly dispersed in ethanol by ultrasonication before the microstructure observation. Therefore, the results obtained via the XRD tests demonstrate that the ZrP, PDA, and AuNPs were integrated into a well-structured composite, which is a prerequisite for obtaining significant catalytic activity for the conversion of 4NP to 4AP.

**Figure 5:**
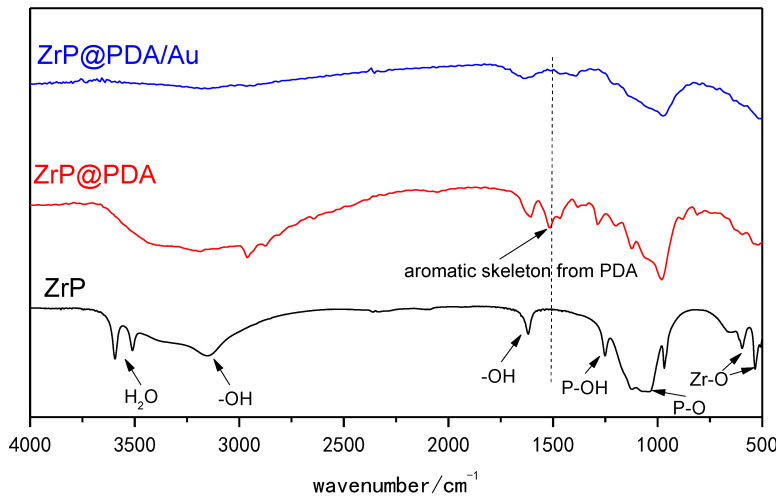
FTIR spectra of ZrP, ZrP@PDA, and ZrP@PDA/Au samples.

FTIR spectra were further employed to clarify the surface functionalities on the typical ZrP, ZrP@PDA, and ZrP@PDA/Au samples (see Figure 5). The typical FTIR absorption bands probed for the present ZrP sample are consistent with those of previous studies [41,42,43]. For example, the typical absorption bands at 3590 and 3500 cm^−1^ stemmed from the interlayer water due to the hydrophilicity of ZrP. The P-O and P-OH groups of the ZrP microcrystals were detected by the adsorption bands at around 1100 and 1250 cm^−1^. The absorption peaks at approximately 530 and 600 cm^−1^ were indexed to the Zr-O bonds of the ZrP microcrystals. The abundant -OH groups of ZrP make it hydrophilic, and the FTIR signals associated with -OH groups and water molecules under different association states were observed at approximately 3150 and 1645 cm^−1^. The surface modification of the ZrP resulted in a new FTIR band at about 1518 cm^−1^, attributable to the aromatic skeletons from the PDA layers on the ZrP surface. The further deposition of ultra-small AuNPs on the ZrP@PDA surface, achieved by PDA-enabled reduction reactions, largely weakened the FTIR band at around 1518 cm^−1^, which might have been due to the participation of the aromatic skeleton in the conversion of the Au ions into AuNPs. In addition, the uniform coating of AuNPs onto the ZrP@PDA surface may have also shielded the FTIR absorption from PDA moiety, thus causing the FTIR signal from the PDA to be weakened. 

**Figure 6:**
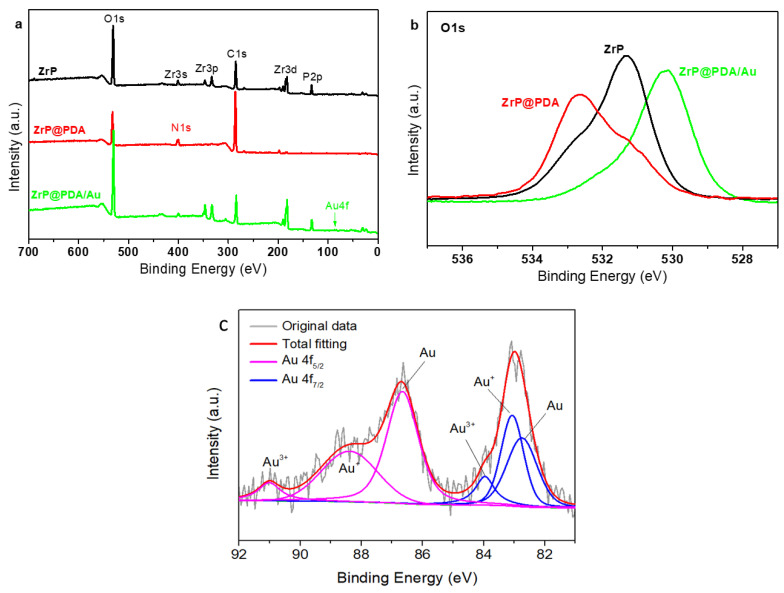
XPS survey (**a**) and high-resolution XPS O1s (**b**) spectra of ZrP, ZrP@PDA, and ZrP@PDA/Au; (**c**) Au 4f spectrum of ZrP@PDA/Au.

The XPS spectra are provided in Figure 6 to clarify the chemical states and composition of the typical samples. The XPS survey spectra shown in Figure 6a present the chemical compositions of the ZrP, ZrP@PDA, and ZrP@PDA/Au samples. The ZrP sample comprised Zr, P, O, and C elements, and the origin of carbon was from the carbonaceous contaminants adsorbed on the ZrP surface. For the ZrP@PDA, along with strengthening the C1s signal, a new signal of N1s emerged, ascribed to PDA, thus verifying the successful modification of the ZrP with the PDA layers. The decoration of the AuNPs onto the ZrP@PDA surface resulted in the signal of Au 4f, stemming from the effective reaction between the PDA and Au ions and the immobilization of the AuNPs onto the adhesive PDA coating. The high-resolution XPS O1s spectra of the typical samples in Figure 6b reveal that the PDA modification caused the O1s peak to shift to the larger binding energies due to the stronger intermolecular interactions between the ZrP substrate and the PDA modifier, such as hydrogen bonding between the -OH groups of the ZrP and the oxygen/nitrogen-containing functional groups of the PDA. The further decoration of the AuNPs onto the ZrP@PDA shifted the O1s peak to lower binding energies, likely attributed to the complexation of the AuNPs with the oxygen/nitrogen group PDA. The high-resolution XPS Au 4f spectrum of the typical ZrP@PDA/Au sample can be deconvoluted into several subpeaks associated with Au^0^, Au^+^, and Au^3+^ (Figure 6c). The generation of Au^+^ and Au^3+^ can be ascribed to the highly reactive surface of the AuNPs. The smaller the AuNPs, the more reactive surface they possess due to the higher possibility of exposing more unsaturated atoms on the corners, steps, and kinks of the smaller AuNPs. 

**Figure 7:**
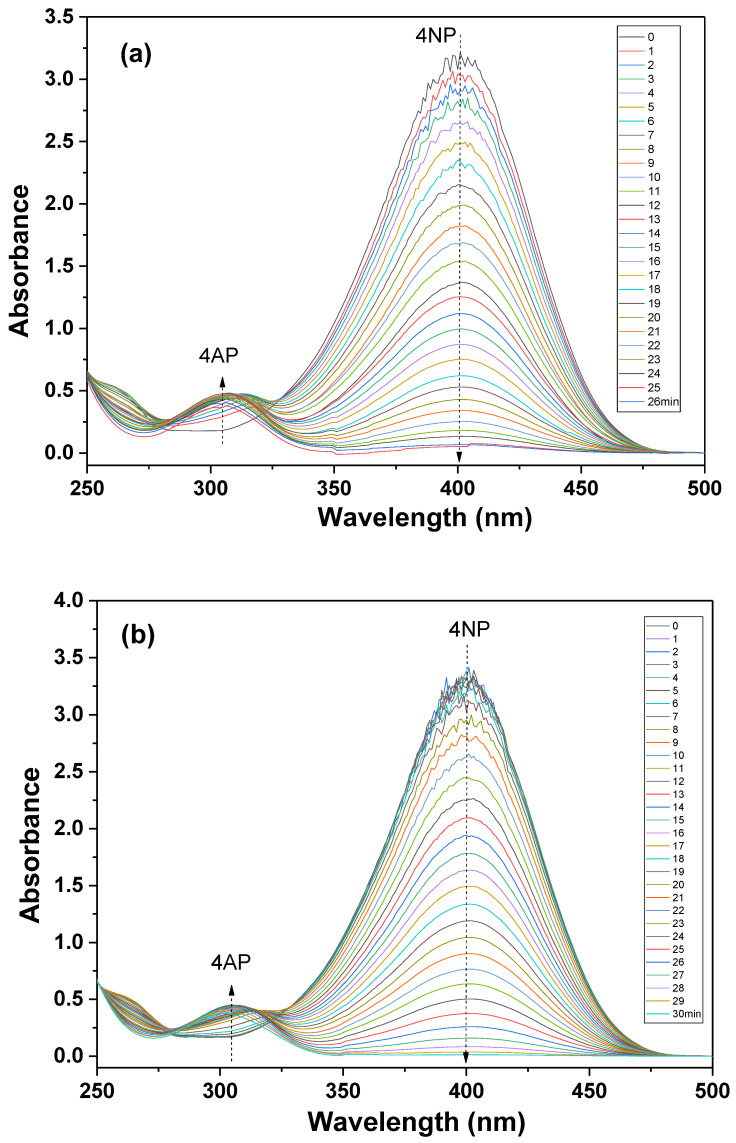

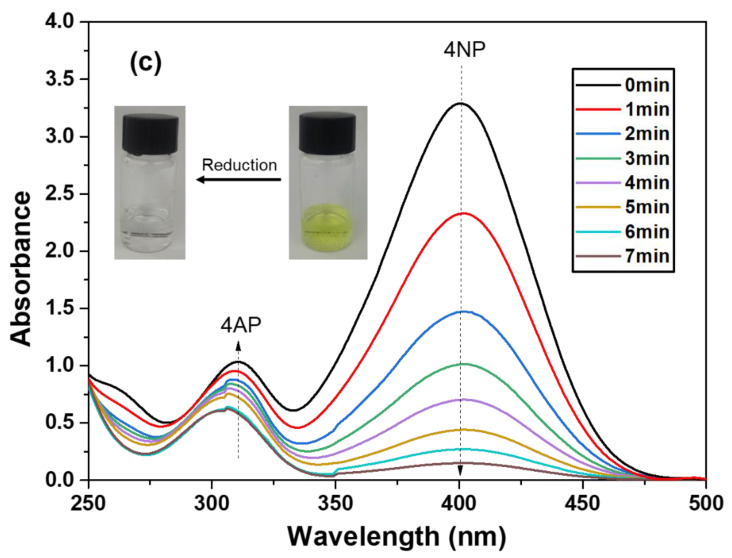
Catalytic conversion of 4NP to 4AP using the (**a**) ZrP/Au, (**b**) PDA/Au and (**c**) ZrP@PDA/Au catalysts.

**Figure 8:**
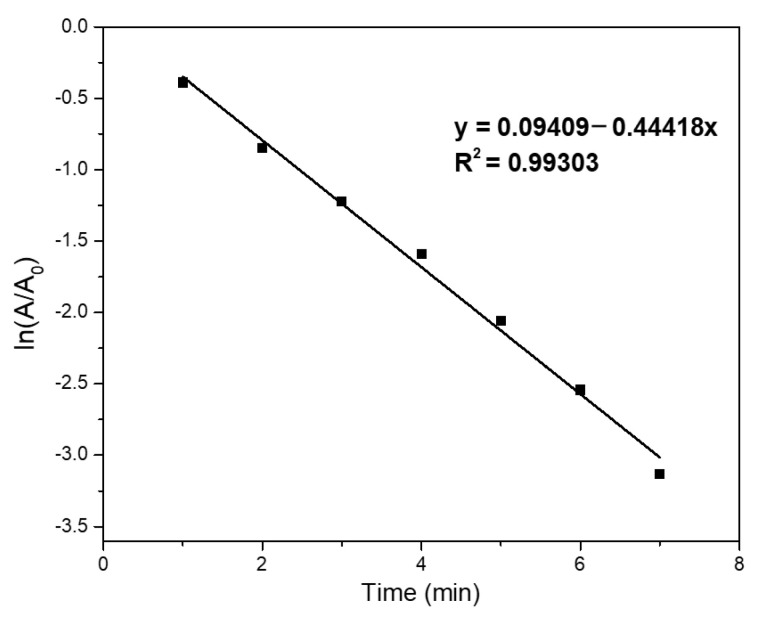
Plots of ln(A_t_/A_0_) as a function of the reaction time for the catalytic reduction of 4NP to 4AP over the prepared ZrP@PDA/Au catalyst.

The catalytic conversion of 4NP to 4AP has been widely adopted as a valuable reaction model to assess the performance of Au-based catalysts due to its easy operation at room temperature with no harsh requirements in the reaction conditions. In addition, the commonly used UV-Vis spectroscopy can monitor such a reaction process. The conversion of 4NP to 4AP can be tracked by a progressional decrease in the intensity of the UV-Vis absorption peak at approximately 400 nm and by gradually strengthening the peak around 300 nm (Figure 7). We first compared the catalytic activities of the typical Au catalyst deposited on different substrates (in this case, ZrP/Au, PDA/Au, and ZrP@PDA/Au). It can be noted that the PDA played a critical role in yielding the high-performance Au-based catalyst. It took around 26 min to complete the reduction of 4NP over the ZrP/Au catalyst (Figure 7a), which is much inferior to the ZrP@PDA/Au counterpart (Figure 7c), capable of a highly efficient conversion of 4NP to 4AP, with only approximately 7 min needed to complete the reaction. Thus, the inclusion of PDA promoted the reaction efficiency by about four times, primarily due to the abundant binding sites provided by the PDA uniformly coated on the ZrP surface. The abundant complexation sites of PDA highly dispersed and immobilized the AuNPs, thus resulting in the ultra-small size of the active Au catalysts and the corresponding larger amount of active Au surface for the catalytic reaction. As for the PDA/Au catalyst, the reaction time of the 4NP reduction was up to 30 min, including an induction period of ca. 5 min (Figure 7b), indicating even less catalytic activity than that of ZrP/Au. Thereby, it can be concluded that the single-layer ZrP nanosheets also contributed significantly to the catalytic activity of the AuNPs, since the highly dispersed nanosheets prevented the aggregation of the PDA and AuNPs. Due to the excessive reducing agent added, the catalytic reduction was assumed to be a pseudo-first-order reaction. The good linear fitting of the catalytic kinetics of the ZrP@PDA/Au-catalyzed reduction of 4NP shown in Figure 8 is consistent with the assumption. One can calculate the apparent reaction rate and TOF based on the linear fitting. The prepared ZrP@PDA/Au catalyst exhibited an impressive TOF (38.10 min^−1^), far outstripping some reported noble metal-based catalysts (2.56~32.36 min^−1^), as summarized in Table 1 [18,44,45,46].

**Figure 9:**
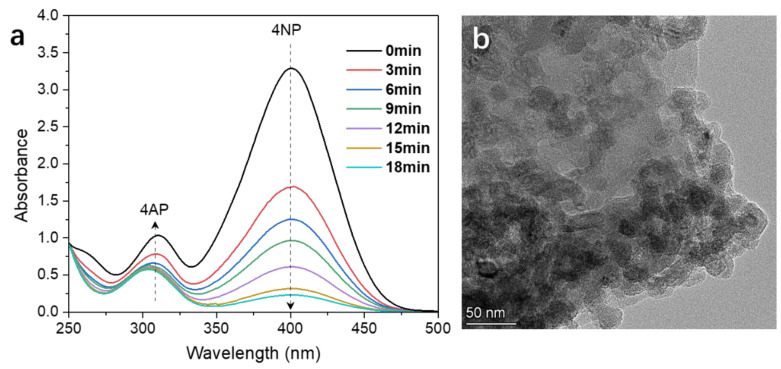
(**a**) Probing the catalytic activity variation after storing the ZrP@PDA/Au catalyst for six days. (**b**) TEM image of the ZrP@PDA/Au catalyst after being stored in solution for six days.

Apart from the catalytic activity, the reusability and stability are also vital for practical applications. Thus, they were tested for the typical ZrP@PDA/Au catalyst, and the result is presented in Figure 9 and Figure S1 in the Supporting Information (SI). The reusability experiments demonstrated that only 3 min were needed for the complete reduction of 4NP to 4AP under stirring conditions for all five cycles (Figure S1 in the SI), thus revealing that the catalytic activity of ZrP@PDA/Au was retained, without any degradation, after the five cycles. On the other side, after prolonged storage of the ZrP@PDA/Au sample in solution for six days, the catalytic efficiency was only lowered to an acceptable level, and a short duration (around 18 min, Figure 9a) was needed for almost completing the reduction of 4NP to 4AP. The decrease in the catalytic efficiency upon the prolonged storage of the ZrP@PDA/Au catalyst can be ascribed to the moderate aggregation of AuNPs in water, indicated by the TEM morphology variation before and after the prolonged storage treatment (Figure 9b). Altogether, the ZrP@PDA/Au catalyst exhibited high activity, reusability, and stability for efficient and durable catalytic transformation reactions.

## 4. Conclusions

We have reported the modification of ZrP via mussel-inspired chemistry based on PDA to provide powerful surface functionalities capable of the straightforward conversion of chloroauric acid into ultra-small AuNPs with an average particle size of approximately 6.5 nm based on TEM images. The PDA was demonstrated to play a critical role in the construction of the ternary ZrP@PDA/Au catalyst, far outperforming the binary ZrP/Au counterpart in the activity toward the transformation of 4NP to 4AP. The high activity of the supported ultra-small AuNPs resulted in a significant TOF as high as 38.10 min^−1^, much superior to those of some reported supported noble metal catalysts. Apart from high catalytic activity, the stability and reusability of the ZrP@PDA/Au were also well demonstrated. Therefore, the study presented here sheds light on decoding the facile and green method of the straightforward fabrication of metal nanocatalysts with a tiny nano-size with a low cost and using biocompatible support materials. 

## Figures and Tables

**Table 1:** Catalytic activities of various supported noble metal nanocatalysts.

Catalyst	Completion Time (s)	*k*_app_ (10^−3^ s^−1^) ^a^	TOF (min^−1^) ^b^	Reference
**ZrP@PDA/Au**	**420**	**7.4**	**38.10**	**this work**
ZrP@PDA/Ag	180	34.76	32.36	[18]
AgNPs/SiNSs	40	80.19	3.52	[44]
AuNPs/SNTs	280	10.64	--	[45]
PLAL-AuNPs/CeO_2_-NTs	1200	2.25	0.367	[47]
Au@MOF-3	60	68.8	0.016	[48]
Au@TR-HCP-TPMT	480	1.67	1.19	[49]

^a^ *k*_app_: apparent rate constant. ^b^ TOF: the turnover frequency, the moles of 4NP reduced per mole of Au (or Ag) per minute.

## Data Availability

Not applicable.

## Supplementary Materials

The following supporting information can be downloaded at: https://www.mdpi.com/article/10.3390/nano12193339/s1, Figure S1: UV-vis absorption spectra of the reduction of 4-NP in the reusability experiments.

## References

[1] Hu H., Wen W., Ou J.Z. (2022). Construction of adsorbents with graphene and its derivatives for wastewater treatment: A review. Environ. Sci.-Nano.

[2] Xu Y., Liang Y., Yuai Z., Long H., He Q., Guo K., Zhang Y., Chen D., Xu X., Hu H. (2022). Co-doping g-C3N4 with P and Mo for efficient photocatalytic tetracycline degradation under visible light. Ceram. Int..

[3] Zhang J., Zhang Y., Huang H., Huang K., Deng L., Hu H., Chang M., Chen D., Wang Y. (2022). Exploration of Highly Porous Biochar Derived from Dead Leaves to Immobilize LiOH·H_2_O Nanoparticles for Efficient Thermochemical Heat Storage Applications. Energy Technol..

[4] Zhang W., Fan T., Yang Z., Yu R., Zeng X., Xu Y., Zhang M., Hu H., Ou J.Z., Zheng L. (2022). Crystal phase-driven copolymerization of CO_2_ and cyclohexene oxide in Prussian blue analogue nanosheets. Appl. Mater. Today.

[5] Zhu W., Lin Y., Kang W., Quan H., Zhang Y., Chang M., Wang K., Zhang M., Zhang W., Li Z. (2020). An aerogel adsorbent with bio-inspired interfacial adhesion between graphene and MoS_2_ sheets for water treatment. Appl. Surf. Sci..

[6] Hu H., Ou J.Z., Xu X., Lin Y., Zhang Y., Zhao H., Chen D., He M., Huang Y., Deng L. (2021). Graphene-assisted construction of electrocatalysts for carbon dioxide reduction. Chem. Eng. J..

[7] Xu X., Huang T., Xu Y., Hu H., Liao S., Hu X., Chen D., Zhang M. (2022). Highly dispersed CeO_2_–nanoparticles with rich oxygen vacancies enhance photocatalytic performance of g-C3N4 toward methyl orange degradation under visible light irradiation. J. Rare Earths.

[8] Wang W.-Y., Chiou J.-C., Chen W.-X., Kan C.-W., Lam T.Y.C., Hu H. (2022). Poly(hexamethylene biguanide): An efficient pH-tolerant and salt-intensive flocculant in the removal of anionic dyes from wastewater. J. Mater. Sci..

[9] Du C., Zhang Y., Zhang Z., Zhou L., Yu G., Wen X., Chi T., Wang G., Su Y., Deng F. (2022). Fe-based metal organic frameworks (Fe-MOFs) for organic pollutants removal via photo-Fenton: A review. Chems. Eng. J..

[10] Zhang J., Wei J., Massey I.Y., Peng T., Yang F. (2022). Immobilization of Microbes for Biodegradation of Microcystins: A Mini Review. Toxins.

[11] Hu H., Chang M., Zhang M., Wang X., Chen D. (2017). A new insight into PAM/graphene-based adsorption of water-soluble aromatic pollutants. J. Mater. Sci..

[12] Goudjil S., Guergazi S., Masmoudi T., Achour S. (2021). Effect of reactional parameters on the elimination of Congo Red by the combination of coagulation-floculation with aluminum sulphate. Desalin. Water Treat..

[13] Hu H., Quan H., Zhong B., Li Z., Huang Y., Wang X., Zhang M., Chen D. (2018). A Reduced Graphene Oxide Quantum Dot-Based Adsorbent for Efficiently Binding with Organic Pollutants. ACS Appl. Nano Mater..

[14] Hu H., Liang W., Zhang Y., Wu S., Yang Q., Wang Y., Zhang M., Liu Q. (2018). Multipurpose Use of a Corncob Biomass for the Production of Polysaccharides and the Fabrication of a Biosorbent. ACS Sustain. Chem. Eng..

[15] Zhang Y., Hu H., Chang M., Wei H., Chen D., Zhang M., Wu L., Li X. (2017). Template-free scalable synthesis of TiO2 hollow nanoparticles for excellent photoelectrochemical applications. J. Mater. Sci..

[16] Zhang Y., Hu H., Kang W., Qiu G., Liang R., Deng L., Yuan H. (2020). Enhancing hydrogen evolution by photoelectrocatalysis of water splitting over a CdS flowers-loaded TiO_2_ nanotube array film on the Ti foil substrate. Ceram. Int..

[17] Hu H., Chang M., Wang X., Chen D. (2017). Cotton fabric-based facile solar photocatalytic purification of simulated real dye wastes. J. Mater. Sci..

[18] Xu Y., Zhou F., Chen M., Hu H., Lin L., Wu J., Zhang M. (2020). Facile assembly of 2D α-zirconium phosphate supported silver nanoparticles: Superior and recyclable catalysis. New J. Chem..

[19] Hu H.-W., Xin J.H., Hu H. (2013). Highly Efficient Graphene-Based Ternary Composite Catalyst with Polydopamine Layer and Copper Nanoparticles. ChemPlusChem.

[20] Hu H., Xin J.H., Hu H. (2014). PAM/graphene/Ag ternary hydrogel: Synthesis, characterization and catalytic application. J. Mater. Chem. A.

[21] Hu H., Xin J.H., Hu H., Wang X. (2015). Structural and mechanistic understanding of an active and durable graphene carbocatalyst for reduction of 4-nitrophenol at room temperature. Nano Res..

[22] Hu H., Wang X., Miao D., Wang Y., Lai C., Guo Y., Wang W., Xin J.H., Hu H. (2015). A pH-mediated enhancement of the graphene carbocatalyst activity for the reduction of 4-nitrophenol. Chem. Commun..

[23] Hu H., Xin J.H., Hu H., Wang X., Kong Y. (2015). Metal-free graphene-based catalyst—Insight into the catalytic activity: A short review. Appl. Catal. A Gen..

[24] Hu H., Xin J.H., Hu H., Wang X., Miao D., Liu Y. (2015). Synthesis and stabilization of metal nanocatalysts for reduction reactions—A review. J. Mater. Chem. A.

[25] Zhang F., Yang P., Matras-Postolek K. (2016). Au Catalyst Decorated Silica Spheres: Synthesis and High-Performance in 4-Nitrophenol Reduction. J. Nanosci. Nanotechnol..

[26] Zhang M., Lu X., Wang H.-Y., Liu X., Qin Y., Zhang P., Guo Z.-X. (2016). Porous gold nanoparticle/graphene oxide composite as efficient catalysts for reduction of 4-nitrophenol. RSC Adv..

[27] Al-Kahtani A.A., Almuqati T., Alhokbany N., Ahamad T., Naushad M., Alshehri S.M. (2018). A clean approach for the reduction of hazardous 4-nitrophenol using gold nanoparticles decorated multiwalled carbon nanotubes. J. Clean. Prod..

[28] Beaton G., Zacks J., Stamplecoskie K. (2022). Al_2_O_3_ anchored silver and gold nanoparticles as accessible, stable, and re-usable catalysts. Colloids Surf. A.

[29] Qu H., Yang L., Yu J., Wang L., Liu H. (2018). Host–Guest Interaction Induced Rapid Self-Assembled Fe_3_O_4_@Au Nanoparticles with High Catalytic Activity. Ind. Eng. Chem. Res..

[30] Dai Y., Yu P., Zhang X., Zhuo R. (2016). Gold nanoparticles stabilized by amphiphilic hyperbranched polymers for catalytic reduction of 4-nitrophenol. J. Catal..

[31] Sun L., Jiang L., Peng S., Zheng Y., Sun X., Su H., Qi C. (2019). Preparation of Au catalysts supported on core-shell SiO_2_/polypyrrole composites with high catalytic performances in the reduction of 4-nitrophenol. Synth. Met..

[32] Obulapuram P.K., Arfin T., Mohammad F., Kumari K., Khiste S.K., Al-Lohedan H.A., Chavali M. (2021). Surface-Enhanced Biocompatibility and Adsorption Capacity of a Zirconium Phosphate-Coated Polyaniline Composite. ACS Omega.

[33] Pica M., Calzuola S., Donnadio A., Gentili P., Nocchetti M., Casciola M. (2018). De-Ethylation and Cleavage of Rhodamine B by a Zirconium Phosphate/Silver Bromide Composite Photocatalyst. Catalysts.

[34] Sanchez J., Stevens M.B., Young A.R., Gallo A., Zhao M., Liu Y., Ramos-Garcés M.V., Ben-Naim M., Colón J.L., Sinclair R. (2021). Isolating the Electrocatalytic Activity of a Confined NiFe Motif within Zirconium Phosphate. Adv. Energy Mater..

[35] Xiao H., Liu S. (2018). Zirconium phosphate (ZrP)-based functional materials: Synthesis, properties and applications. Mater. Des..

[36] Campoccia D., Ravaioli S., Vivani R., Donnadio A., Vischini E., Russo A., Visai L., Arciola C.R., Montanaro L., Nocchetti M. (2019). Antibacterial Properties of a Novel Zirconium Phosphate-Glycinediphosphonate Loaded with Either Zinc or Silver. Materials.

[37] Sun L., Boo W.J., Sue H.-J., Clearfield A. (2007). Preparation of alpha-zirconium phosphate nanoplatelets with wide variations in aspect ratios. New J. Chem..

[38] Xu Y., Lin L., Zeng S., Liu J., Xiao M., Wang S., Meng Y., Sun L. (2019). Synthesis of Polylactide Nanocomposites Using an α-Zirconium Phosphate Nanosheet-Supported Zinc Catalyst via in Situ Polymerization. ACS Appl. Polym. Mater..

[39] Shi Z., Tang J., Chen L., Yan C., Tanvir S., Anderson W.A., Berry R.M., Tam K.C. (2015). Enhanced colloidal stability and antibacterial performance of silver nanoparticles/cellulose nanocrystal hybrids. J. Mater. Chem. B.

[40] Cheng Y., Wang X.T., Jaenicke S., Chuah G.-K. (2017). Minimalistic Liquid-Assisted Route to Highly Crystalline α-Zirconium Phosphate. ChemSusChem.

[41] Thakkar R., Patel H., Chudasama U. (2007). A comparative study of proton transport properties of zirconium phosphate and its metal exchanged phases. Bull. Mater. Sci..

[42] Zhou Y., Wang A., Wang Z., Chen M., Wang W., Sun L., Liu X. (2015). Titanium functionalized [small alpha]-zirconium phosphate single layer nanosheets for photocatalyst applications. RSC Adv..

[43] Zhou Y., Huang R., Ding F., Brittain A.D., Liu J., Zhang M., Xiao M., Meng Y., Sun L. (2014). Sulfonic acid-functionalized alpha-zirconium phosphate single-layer nanosheets as a strong solid acid for heterogeneous catalysis applications. ACS Appl. Mater. Interfaces.

[44] Yan Z., Fu L., Zuo X., Yang H. (2018). Green assembly of stable and uniform silver nanoparticles on 2D silica nanosheets for catalytic reduction of 4-nitrophenol. Appl. Catal. B Environ..

[45] Zhang Z., Shao C., Zou P., Zhang P., Zhang M., Mu J., Guo Z., Li X., Wang C., Liu Y. (2011). In situ assembly of well-dispersed gold nanoparticles on electrospun silica nanotubes for catalytic reduction of 4-nitrophenol. Chem. Commun..

[46] Mao H., Ji C., Liu M., Cao Z., Sun D., Xing Z., Chen X., Zhang Y., Song X.-M. (2018). Enhanced catalytic activity of Ag nanoparticles supported on polyacrylamide/polypyrrole/graphene oxide nanosheets for the reduction of 4-nitrophenol. Appl. Surf. Sci..

[47] Zhang J., Chen G., Chaker M., Rosei F., Ma D. (2013). Gold nanoparticle decorated ceria nanotubes with significantly high catalytic activity for the reduction of nitrophenol and mechanism study. Appl. Catal. B Environ..

[48] Gole B., Sanyal U., Mukherjee P.S. (2015). A smart approach to achieve an exceptionally high loading of metal nanoparticles supported by functionalized extended frameworks for efficient catalysis. Chem. Commun..

[49] He J., Razzaque S., Jin S., Hussain I., Tan B. (2019). Efficient Synthesis of Ultrafine Gold Nanoparticles with Tunable Sizes in a Hyper-Cross-Linked Polymer for Nitrophenol Reduction. ACS Appl. Nano Mater..

