# Supplementary material for: Mussel-Inspired Surface Modification of α-Zirconium Phosphate Nanosheets for Anchoring Efficient and Reusable Ultrasmall Au Nanocatalysts
Source: Nanomaterials (Basel). 2022 Sep 25;12(19):3339. doi: 10.3390/nano12193339 (PMC9565343; doi:10.3390/nano12193339)
Supplement: Supplementary file 1 [file nanomaterials-12-03339-s001.zip › nanomaterials-1919825-supplementary.pdf]

Supporting Information

Mussel-Inspired Surface Modification of α-Zirconium Phosphate Nanosheets for Anchoring Efficient and Reusable Ultrasmall Au Nanocatalysts

Limiao Lin[1,†], Yi Wen[2,†], Lixi Li[2], Ying Tan[2], Peng Yang[2], Yaoheng Liang[2],

Yisheng Xu[2], Huawen Hu[2,*], Yonghang Xu[2,*]

[1]*School of Environmental and Chemical Engineering, Foshan University, Foshan 528000, China*

[2]*School of Materials Science and Hydrogen Energy, Foshan University, Foshan 528000, China*

†These authors contributed equally to this work.

*Corresponding authors: *huawenhu@126.com (H.H.); yonghangxu@fosu.edu.cn (Y.X.)*

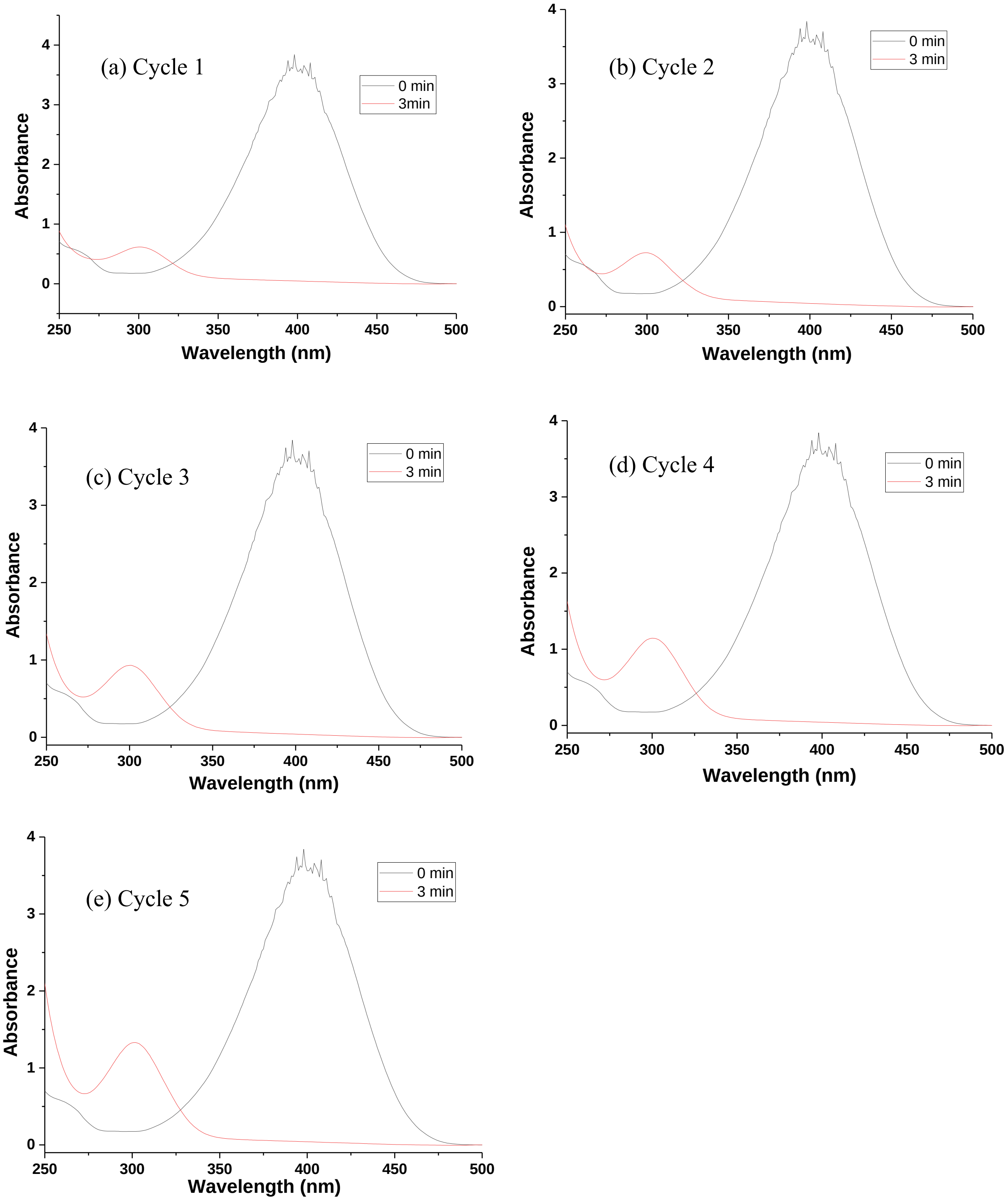

Figure S1. UV-vis absorption spectra of the reduction of 4-NP in the reusability experiments.
